# Tumor evolutionary trajectories during the acquisition of invasiveness in early stage lung adenocarcinoma

**DOI:** 10.1038/s41467-020-19855-x

**Published:** 2020-11-27

**Authors:** Siwei Wang, Mulong Du, Jingyuan Zhang, Weizhang Xu, Qianyu Yuan, Ming Li, Jie Wang, Hongyu Zhu, Yuzhuo Wang, Cheng Wang, Yuhua Gong, Xiaonan Wang, Zhibin Hu, David C. Christiani, Lin Xu, Hongbing Shen, Rong Yin

**Affiliations:** 1 Department of Thoracic Surgery, Jiangsu Key Laboratory of Molecular and Translational Cancer Research, Nanjing Medical University Affiliated Cancer Hospital & Jiangsu Cancer Hospital & Jiangsu Institute of Cancer Research, 21009 Nanjing, P.R. China; 2 Department of Biostatistics, Center for Global Health, School of Public Health, Nanjing Medical University, 211116 Nanjing, P.R. China; 3 Department of Environmental Health, Harvard T. H. Chan School of Public Health, Boston, MA 02115 USA; 4 Department of Epidemiology, Harvard T. H. Chan School of Public Health, Boston, MA 02115 USA; 5 Department of Medicine, Massachusetts General Hospital/Harvard Medical School, Boston, MA 02115 USA; 6 Department of Pathology, Nanjing Medical University Affiliated Cancer Hospital & Jiangsu Cancer Hospital & Jiangsu Institute of Cancer Research, 21009 Nanjing, P.R. China; 7 Department of Science and Technology, Nanjing Medical University Affiliated Cancer Hospital & Jiangsu Cancer Hospital & Jiangsu Institute of Cancer Research, 21009 Nanjing, P.R. China; 8 Biobank of Lung Cancer, Jiangsu Biobank of Clinical Resources, 21009 Nanjing, P.R. China; 9 Department of Epidemiology and Biostatistics, International Joint Research Center on Environment and Human Health, Center for Global Health, School of Public Health, Nanjing Medical University, 211116 Nanjing, P.R. China; 10 Geneplus-Beijing Institute, 102206 Beijing, P.R. China; 11 Geneseeq-Nanjing Technology Inc., 210032 Nanjing, P.R. China; 12 Collaborative Innovation Center for Cancer Personalized Medicine, Nanjing Medical University, 211116 Nanjing, P.R. China

**Keywords:** Cancer genomics, Non-small-cell lung cancer, Preclinical research, Non-small-cell lung cancer

## Abstract

The evolutionary trajectories of early lung adenocarcinoma (LUAD) have not been fully elucidated. We hypothesize that genomic analysis between pre-invasive and invasive components will facilitate the description of LUAD evolutionary patterns. We micro-dissect malignant pulmonary nodules (MPNs) into paired pre-invasive and invasive components for panel-genomic sequencing and recognize three evolutionary trajectories. Evolutionary mode 1 (EM1) demonstrates none of the common driver events between paired components, but another two modes, EM2A and EM2B, exhibit critical private alterations restricted to pre-invasive and invasive components, respectively. When ancestral clones harbor *EGFR *mutations, truncal mutation abundance significantly decrease after the acquisition of invasiveness, which may be associated with the intratumoral accumulation of infiltrated B cells. Harboring *EGFR* mutations is critical to the selective pressure and further impacts the prognosis. Our findings extend the understanding of evolutionary trajectories during invasiveness acquisition in early LUAD.

## Abstract (web summary)

Invasive early stage lung adenocarcinoma has a heterogeneous prognosis. Here, the authors microdissect malignant pulmonary nodules to invasive and preinvasive components and study the mutations that are common or private between the lesions, allowing them to understand the evolutionary path of the tumours.

## Introduction

Lung adenocarcinoma (LUAD) is the most commonly diagnosed subtype of lung cancer and the leading cause of cancer deaths, both globally and in China^1^. Although high-resolution computed tomography (CT) screening has resulted in a drastic increase in malignant pulmonary nodules (MPNs)^2^, LUAD is still considered a heterogeneous prognosis disease, even in the early stage^3^. Tumor invasive status has a notable impact on prognosis for LUAD, especially in early-stage cases^4^. Although surgical resection was reported to yield an almost 100% 5-year survival rate for pre-invasive status (AAH, atypical adenomatous hyperplasia and AIS, adenocarcinoma in situ), early invasive LUAD had a worse prognosis with a certain recurrence rate^5^.

The invasive components in MPNs, including minimally invasive adenocarcinoma (MIA) and invasive adenocarcinoma (IAC), are demonstrated to be an important precise prognostic discriminator and better than the T descriptor of the TNM staging system^6,7^. However, there is still little understanding of the initiation, early progression, and evolutionary patterns of invasive components in MPNs^8,9^. Genome-wide somatic mutation analysis has advanced our understanding of critical molecular events in cancer progression and evolution. Although it was proposed that AAH may progress to AIS, MIA and, eventually, IAC in a linear manner^5^, the evolutionary trajectory from pre-invasive to invasive LUAD has not been fully elucidated^10^. Previous studies demonstrated significant genetic differences among AAH, AIS, and MIA; however, pre-invasive and invasive LUAD have never been investigated within a single MPN^10–14^.

*EGFR* and *KRAS* are two frequently mutated driver genes of LUAD. Early-stage *EGFR*-mutated non-small-cell lung cancer (NSCLC) cases usually have a better prognosis than wild-type or *KRAS*-mutated cases, even without tyrosine-kinase inhibitor (TKI) application^15–17^. Additionally, *EGFR* mutation is considered a positive prognostic marker of both disease-free survival (DFS) and overall survival (OS) in T_1-2a_N_0_M_0_ patients without adjuvant and TKI treatments^15^. This evidence implies that *EGFR* mutations may contribute differently to clonal selection in early-stage LUAD evolution compared to *KRAS* and other mutations^18^. However, to date, little is known regarding whether and how these dominant driver genes affect early progression from pre-invasive to invasive LUAD.

To delineate the driver molecular events and early invasive progression in MPNs, we included 53 T1 stage LUAD cases of the *ChiCTR1900022521* cohort of Jiangsu Cancer Hospital (JSCH) with micro-dissection and panel-genomic-sequencing methods, as well as 496 T1 stage patients with long-term follow-up from the Boston Lung Cancer Study (BLCS) cohort. We focused on genetic heterogeneities between pre-invasive and invasive components, early invasive patterns, and the prognosis of MPN patients. Phylogenetic analyses showed the differences among evolutionary trajectories, and the results further elucidated strong selective pressure and enhanced B cell infiltration during invasiveness acquisition of MPNs harboring truncal *EGFR* mutations.

## Results

### Study workflow and genetic landscape

**Fig. 1:**
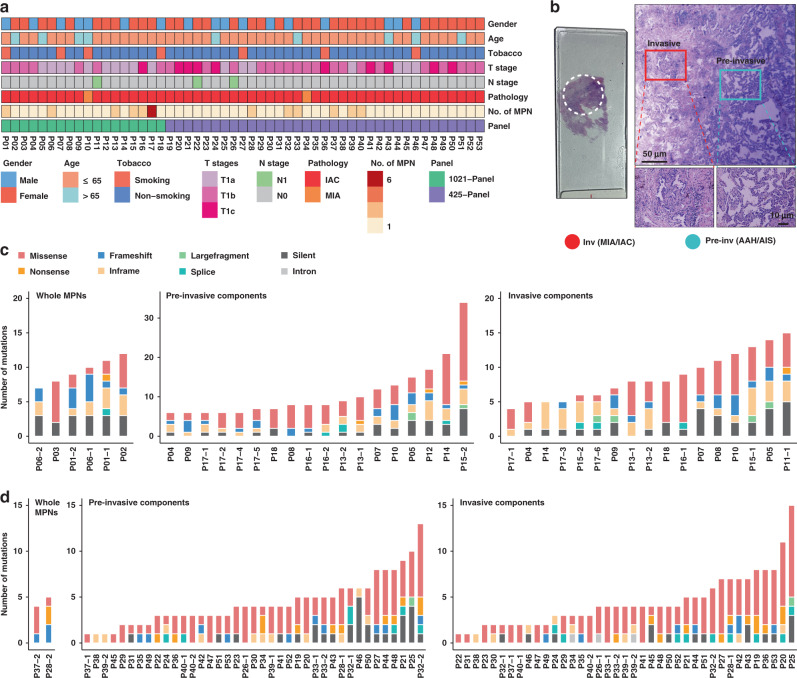
Clinicopathological characteristics and genomic sequencing of micro-dissected MPNs. **a** Clinicopathologic characteristics of the included 53 T1 stage LUAD patients. **b** Micro-dissection was performed to separate pre-invasive and invasive components within MPNs. **c**, **d** Number and type of somatic mutations detected in 6 whole MPNs and 35 micro-dissected MPN components of the cases using 1021-panel (Phase 1; **c**), and somatic mutations detected in 2 whole MPNs and 78 micro-dissected MPN components of the cases using 425-panel (Phase 2; **d**). Inv Invasive, Pre-Inv pre-invasive, AAH atypical adenomatous hyperplasia, AIS adenocarcinoma in situ, MIA minimally invasive adenocarcinoma, IAC invasive adenocarcinoma.

A total of 53 cases were included for the genomic sequencing and data analyses (Supplementary Fig. 1a). All 53 LUAD patients were diagnosed with MPN ≤ 3 cm and pathologically confirmed adenocarcinoma, and lymph node metastasis was found in three cases (Fig. 1a; Supplementary Data 1). Sixty-one of 69 MPNs were conducted with microdissection to separate pre-invasive and adjacent invasive components, including 52 paired components (Supplementary Fig. 1b; Supplementary Data 2). Out of sequenced the 113 MPN components, 8 whole MPNs, 5 metastatic lymph nodes (MLNs), and 79 cfDNA specimens, somatic mutations were found in all MPNs and MLNs and in 23 cfDNA samples (Supplementary Fig. 2a**;** Supplementary Data 3).

Globally, 1–34 somatic mutations (median: 8) were identified in each MPN component of the phase 1 study (Fig. 1c), and 1–15 somatic mutations (median: 4) were observed in the subsequent phase 2 study (Fig. 1d). In addition to the most frequently mutated gene *EGFR*, *TP53*, *MED12*, and *ERBB2* were the top mutated driver genes in tissue samples (Supplementary Fig. 2b). *EGFR* L858R was to be the most recurrent variation in this cohort (Supplementary Fig. 2c), and *EGFR* had higher mutation rates in female cases (Supplementary Fig. 3a). Notably, no significant differences in these driver genes were observed between pre-invasive and invasive MPN components (Supplementary Fig. 3a). The proportions of all six mutation groups showed a greater proportion of C > G transversions in the invasive components (Supplementary Fig. 3b).

### Phylogenetic analyses within MPNs revealed three evolutionary trajectories

**Fig. 2:**
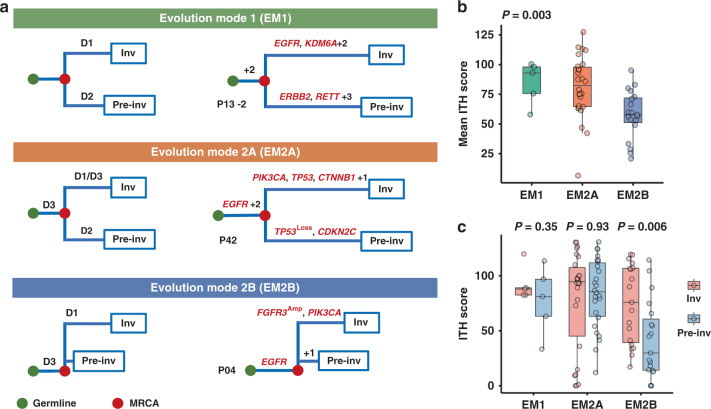
Phylogenetic analyses within MPNs. **a** Evolutionary trajectories of pre-invasive and adjacent invasive MPN components. For each of the three evolutionary patterns, D1, D2, and D3 indicate three hypothetical key molecular events in trunk and branches of phylogenetic tree; green dots represent the germline and red dots represent the most recent common ancestor (MRCA) for each pair of components. In evolution mode 1 (EM1), none of driver mutations are shared and D1 and D2 indicate different private driver alterations (Supplementary Fig. 4a), and JSCH P13-2 is a representative MPN. In evolution mode 2 (EM2), MRCA harbors critical common events (D3). Private driver alterations of D2 and D1 are restricted to the pre-invasive component (EM2A; Supplementary Fig. 4b) and the invasive component (EM2B; Supplementary Fig. 4c), respectively. Phylogenetic trees of JSCH P42 and P04 are shown to represent EM2A and EM2B separately. **b** Mean intratumor heterogeneity (ITH) score of 50 paired pre-invasive and adjacent invasive components suggests phenotypic differences including EM1 (MPNs, *n* = 5), EM2A (MPNs, *n* = 26), and EM2B (MPNs, *n* = 19). The differences were assessed using Kruskal–Wallis *H* test. **c** Comparisons of ITH levels between 50 paired pre-invasive and invasive components were performed using two-sided Wilcoxon Rank-Sum test. The box plot displays the first and third quartiles (top and bottom of the boxes), median (band inside the boxes), and lowest and highest point within 1.5 times the interquartile range of the lower and higher quartile (whiskers). Inv, Invasive and Pre-Inv, Pre-invasive.

To investigate the evolutionary relationship between early pre-invasive and invasive LUAD within the MPNs, we analyzed on 52 paired pre-invasive and adjacent invasive MPN components using phylogenetic methods (Fig. 2a; Supplementary Fig. 4**;** Supplementary Data 4). First, five MPNs from three cases revealed no truncal driver mutations between pre-invasive and adjacent invasive components (Supplementary Fig. 4a), which indicated that pre-invasive and invasive LUAD were driven by different driver events in this situation (Evolution Mode 1, EM1). Second, a total of 45 MPNs harbored truncal critical alterations between pre-invasive and adjacent invasive components (Evolution Mode 2, EM2), and we detected key mutations restricted to pre-invasive branches in 26 of these MPNs, which were classified as EM2A (Supplementary Fig. 4b). In the remaining 19 pairs we observed branch driver mutations only in the invasive components, which revealed a potential linear progression for pre-invasive and adjacent invasive LUAD (classified as EM2B; Supplementary Fig. 4c). Third, no private mutations were discovered in the remaining two MPNs (Supplementary Fig. 4d), which suggests the limitation of our approach to detect other potential key alterations in branches. We also performed phylogenetic analyses of the pre-invasive, invasive components, and MLNs in JSCH P26 of EM2B, which buttressed the supposed linear evolution (Supplementary Fig. 4e).

To estimate genetic relatedness, we quantified the intratumor heterogeneity (ITH) of paired components within each evolution mode. The results demonstrated that EM1 got the highest ITH level, and EM2B had the lowest ITH level, as expected (Fig. 2b**;** Supplementary Fig. 5a). The ITH level of invasive components was significantly higher than the adjacent pre-invasive components in EM2B (Fig. 2c; Supplementary Fig. 5b), which indicates clonal expansion during the early progression^19^. Notably, we observed differential tumor sizes among EMs (Supplementary Fig. 5c), and we supposed that a strong interclonal competition of EM1 would lead to a relatively smaller tumor size compared with EM2.

### Dominant driver genes in truncal mutations

**Fig. 3:**
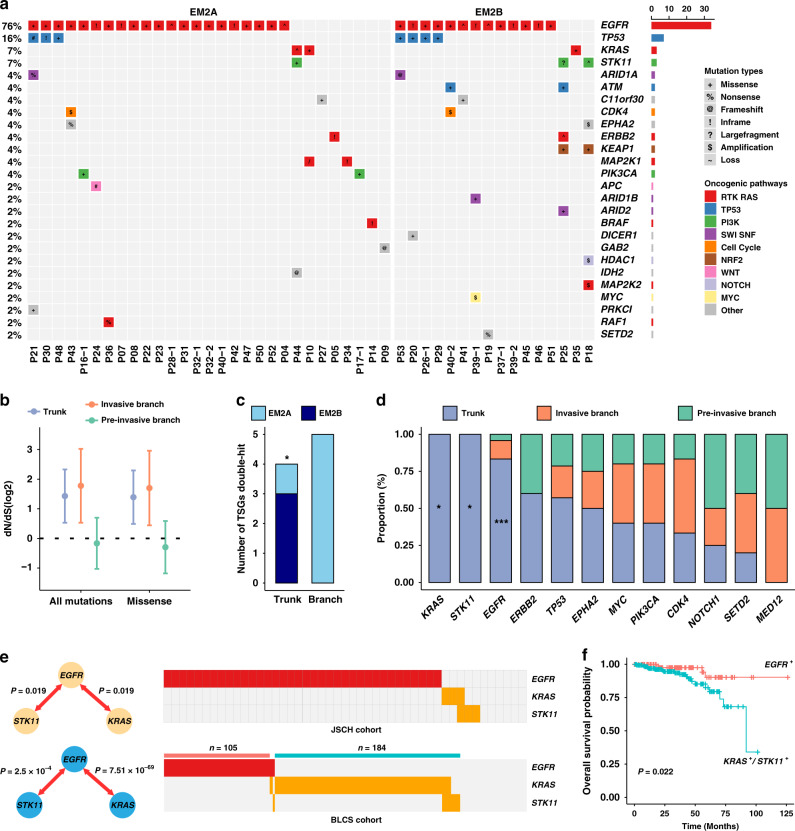
Dominant drivers in truncal genes are associated with clinical outcomes. **a** Mutational spectrum of recurrent non-synonymous truncal mutations. Truncal genes were defined according to known oncogenic pathways identical to those previously reported (see “Methods” section). **b** The ratios of nonsynonymous to synonymous mutations among trunks, pre-invasive, and invasive branches among all MPNs (*n* = 52). The d*N*/d*S* ratios of all nonsynonymous mutations or missense mutations relative to synonymous mutations are shown (on log2 scale). Circles and vertical lines correspond to the mean and 95% confidence intervals of the d*N*/d*S* ratio, respectively. **c** Differences in tumor suppressor gene (TSG) double-hit events, including gene loss, homozygotic mutation and LOH plus mutation, between EM2A (MPNs, *n* = 26) and EM2B (MPNs, *n* = 19) were compared using two-sided Fisher’s exact test (*P* = 0.048). **d** The proportions of truncal and branching mutations in each of the recurrent driver genes of EM2 (MPNs, *n* = 45). Corresponding *P* values calculated by two-sided Fisher’s exact test in *KRAS*, *STK11*, and *EGFR* were 0.035, 0.035, and 6.96 × 10^-8^, respectively. **e** Mutually exclusive analyses of truncal mutations in the JSCH cohort and driver mutations in the BLCS cohort, which suggest two typical clusters of LUAD patients. *P* value was calculated using pair-wise Fisher’s exact test. **f** Kaplan–Meier curves using the BLCS cohort to compare the prognosis between *EGFR-* and *KRAS/STK11-*mutated T1 stage cases in BLCS cohort. *P* value was calculated using log-rank test is indicated. ***P < 0.001; *P < 0.05.

The truncal mutations profile indicated that *EGFR*, *TP53*, *KRAS*, and *STK11* were recurrently mutated driver genes, and almost all truncal mutation genes were known drivers (Fig. 3a). *EGFR*, *CDK4*, and *TP53* were the top frequently altered driver genes of the invasive branching mutations (Supplementary Fig. 6a), and *TP53* carried the highest number of alterations in the pre-invasive branch mutation profile (Supplementary Fig. 6b). We also found that RTK-RAS pathway-related genes (i.e., *EGFR*, *KRAS*, and *ERBB2*) contributed mostly to truncal mutations both in two-phase studies (Supplementary Fig. 6c). As expected, the decreased ratio of pre-invasive branching mutations (d*N*/d*S* ratio) suggested a relaxed ability of promoting progression in pre-invasive branches (Fig. 3b; Supplementary Fig. 6d). We also considered the potential function of tumor suppressor genes (TSGs) in affecting tumor evolution^20^, and the results demonstrated that critical double-hit events of TSGs, including gene loss, homozygotic mutation, and loss of heterozygosity (LOH) plus mutation, contributed differentially to EM2A and EM2B (Fig. 3c; Supplementary Data 6).

*EGFR*, *KRAS*, and *STK11* were three dominant truncal driver genes (Fig. 3d). Somatic interaction analysis indicated that *EGFR* was mutually exclusive from the other two truncal drivers, which was validated in the BLCS and TCGA cohorts (Fig. 3e**;** Supplementary Fig. 6e**;** Supplementary Data 7). Importantly, survival analysis indicated a better prognosis of *EGFR*-mutated patients than *KRAS/STK11*-mutated patients in the BLCS cohort (Fig. 3f), and the TCGA data suggested a consistent trend (Supplementary Fig. 6f).

### Truncal *EGFR* mutation is associated with strong selective pressure and B cell infiltration

**Fig. 4:**
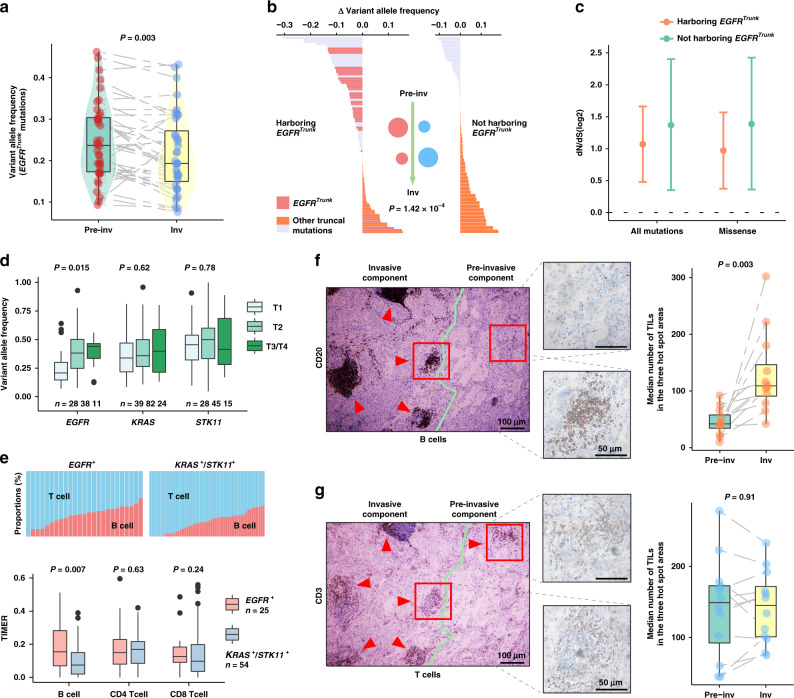
Strong selective pressure derived from B cell infiltration in MPNs harboring truncal *EGFR* mutation. **a** Variant allele frequency (VAF) of identified truncal *EGFR* mutations in 36 paired pre-invasive and adjacent invasive components. Differences were assessed using the two-sided Wilcoxon rank-sum test. **b** Mutant abundance change of identified truncal mutations between two components in EM2, according to whether they harbored truncal *EGFR* mutations. Red and blue circles represent the putative truncal clone abundance of two groups during the invasive progression, respectively. *P* value was derived from two-sided Wilcoxon rank-sum test. **c** The d*N*/d*S* ratios inferred for 36 MPNs harboring and 11 MPNs not harboring truncal *EGFR* mutations. These ratios were obtained as described for Fig. 3b. Circles and vertical lines correspond to the mean and 95% confidence intervals of the d*N*/d*S* ratio, respectively. **d** Mutation abundance in *EGFR*, *KRAS*, and *STK11* mutations in the TCGA LUAD data for patients of stages T1–4. Differences among stages were assessed by Kruskal–Wallis *H* test. **e** Comparisons of T cells and B cells between *EGFR-* and *KRAS/STK11*-mutated groups using TIMER inflammatory infiltration in T1 stage cases of TCGA. *P* value, two-sided Wilcoxon rank-sum test. **f**, **g** Representative sliced IHC images of B cells **f** and T cells **g** in 12 *EGFR*-mutated patients. Two-sided Wilcoxon rank-sum test was used for paired invasive and pre-invasive components. Bar, median; box, 25th–75th percentiles (interquartile range, IQR); vertical line, data within 1.5 times the IQR. Inv Invasive and Pre-inv Pre-invasive.

We compared the abundance of identified truncal *EGFR* mutations between pre-invasive and adjacent invasive components. Intriguingly, the results demonstrated that the abundance of *EGFR* mutations in the invasive component was significantly lower than that in the adjacent pre-invasive component (Fig. 4a). We subsequently analyzed the abundance change of truncal mutations between MPNs harboring or not harboring truncal *EGFR* mutations, and the results suggested that truncal mutation abundance in *EGFR*-mutated MPNs was significantly reduced (Fig. 4b). These results indicated a strong selective pressure on *EGFR*-mutated tumor cells during the acquisition of invasiveness. The decreased d*N*/d*S* ratios of mutations in the MPNs harboring truncal *EGFR* mutation buttressed these findings (Fig. 4c; Supplementary Fig. 6d).

Our previous study observed an association between *EGFR* mutation and inflammatory infiltration in NSCLC patients^21^. Therefore, we proposed that the selective pressure from inflammatory infiltration contributed to the differential prognosis in T1 stage LUAD patients. We analyzed the TCGA cohort and found that the mutation abundance of *EGFR* was significantly decreased in T1 stage, which was different from the exclusive driver genes *KRAS* and *STK11* (Fig. 4d). Additionally, *EGFR* mutation was found to be associated with specific high B cell infiltration in the T1 stage (Fig. 4e; Supplementary Fig. 7c,d). To validate this hypothesis, we further performed immunohistochemistry (IHC) assays to evaluate the microenvironmental B and T cell infiltration of *EGFR*-mutated cases. The results confirmed that B cells (CD20+) were present at higher levels in the invasive component than the adjacent pre-invasive component (Fig. 4f), while no significant differences of T cells (CD3+) between the two components were observed in the serial histological sections (Fig. 4g).

In addition, we identified somatic mutations in 12 prior-operation and 11 post-operation cfDNAs (Supplementary Fig. 8a, b), and no significant differences in mutation abundances were found between prior-operation and post-operation cfDNA samples (Supplementary Fig. 8c). Notably, we did not detect *EGFR* mutations both in prior-operation and post-operation cfDNAs (Supplementary Fig. 8d; Supplementary Data 8).

## Discussion

There is little understanding of the histological continuum preceding early invasive progression in LUAD. Multi-region sampling and genomic sequencing revealed significant ITH, even in the pre-invasive AIS^14,22^. Evaluating the evolutionary trajectory of early invasive LUAD is critical to elucidate the mechanism of early invasive progression, classify molecular genotypes, and provide potential strategies for early intervention. However, conflicting findings were obtained in investigations of early invasive driver events^10,11,14,22^. Evgeny et al. found that *KRAS*, *TP53*, and *EGFR* mutations were indicators of malignant transition from AAH to AIS/MIA^22^. Sivakumar et al. demonstrated that *BRAF* and *KRAS* were initiated as driver events in AAH, but *EGFR* and *TP53* were secondary driver events in LUAD^11^. Xin et al. observed truncal *EGFR* and *KRAS* mutations between pre-invasive and invasive LUAD in multifocal MPNs from the same patients^10^. Zhang et al. demonstrated that *EGFR*, *ERBB2*, *NRAS*, and *BRAF* were early clonal genomic events in AIS, but *TP53* was only found in MIA and IAC^14^. Consistent with previous studies, we revealed that trunk mutations of common driver genes (i.e., *EGFR*, *TP53*, *KRAS*, *and STK11*) played a dominant role in early invasive LUAD. Although we did not observe different proportions of driver genes between pre-invasive and invasive components, driver mutations have direct effects on different evolution trajectories. A greater proportion of C>G transversions was found in invasive components than in pre-invasive components, which indicated potential differences in mutation signatures.

Evolution is always branched^23,24^, and our results demonstrated that pre-invasive and adjacent invasive LUAD arose from branching evolution in 62% (31 in 50) of MPN samples (EM1 and EM2A). As expected, 19 MPNs (EM2B) demonstrated the linear evolution model, which suggests a canonical early invasive progression of the stepwise process from preneoplasia to IAC^5^. To the best of our knowledge, invasive progression in a single lesion was never investigated. Performing a thorough phylogenetic analysis within the MPN is important to demonstrate the evolutionary process from pre-invasive to invasive LUAD. Based on the micro-dissection, we investigated genomic relationships between pre-invasive and invasive components in one MPN. Previous reports revealed significant ITH in AAH/AIS, and the results implied a trend of branched evolution^10,25^. The results of the present study indicated that pre-invasive and invasive components were mostly evolutionary results of branched evolution, which support the findings of the previous studies^10,22^. Additionally, we noted that either pre-invasive or invasive branch was not detected in JSCH P38 and P49, which suggests that our approach could not detect additional differences, such as rare genetic events, epigenetic alterations, and tumor microenvironment infiltration^11^. All of these findings indicated a high complexity of invasive progression in early stage LUAD.

Notably, the *EGFR* dual hotspot variants were found in MPNs JSCH P33-1 and P33-2 of EM1. These findings revealed that dual *EGFR* hotspot mutations derived from pre-invasive and invasive components separately in early invasive LUAD, which may explain the biology of this rare *EGFR* mutation distribution^26^. Approximately 80% (36 in 45) of trunks in EM2 contained *EGFR* exons 19, 20, and 21 variants (Fig. 3a), suggesting a dominant role of *EGFR* variants in LUAD initiation. In addition to high mutation frequency, tumor clones harboring *EGFR* mutations interacted with the tumor microenvironment^21^, even in AAH^10^. Truncal *EGFR* mutations exhibited significantly lower mutation abundance in the invasive component compared to the pre-invasive component within the same MPN. According to the analysis results, we hypothesized that tumor ancestors harboring *EGFR* mutations would undergo negative selective pressure from B cell infiltration during the acquisition of invasiveness. We suppose that indeterminate B-cell-derived cytokines contribute to this biological process^27^. Furthermore, this tumor microenvironment cross-talk may provide an explanation for the inefficiency in detecting *EGFR* mutations in cfDNA samples in this T1 stage cohort.

Our research results serve to elucidate the relationship among genetic heterogeneity, tumor evolution, and long-term prognosis in early invasive LUAD. The major limitation of this study was the hotspot sequencing method, which limited the depiction of comprehensive genomic alterations. Whole exome or genome sequencing and a larger sample size in micro-dissection are needed for follow-up research. Importantly, the mechanism of evolutionary selection during the acquisition of invasiveness warrants further research.

## Methods

### Patient samples and study design

Patients enrolled in this study belonged to a cohort study (A Non-Interventional Systematic Study for the NSCLC Tempo-spatial Heterogeneity; ChiCTR1900022521, http://www.chictr.org.cn/showproj.aspx?proj=34204)^28^. Fifty-three cases were pathologically confirmed as T1 stage LUAD with MPN ≤ 3 cm (American Joint Committee on Cancer, AJCC, 8th edition; clinical data available in Supplementary Data 1). Two pathologists identified and reviewed all patient samples to characterize the histopathological features, and a total of 61 MPNs were micro-dissected under the stereomicroscope to separate pre-invasive and invasive components. The Institutional Review Board of JSCH approved the study, and all the patients provided written informed consent.

In the phase 1 study, 80 specimens of the first 18 cases were subjected to wide panel-genomic sequencing (pan-cancer 1021-gene panel, Geneplus Technology Inc.) at the coverage depth of 1800×. In the phase 2 study, 125 specimens of 35 cases were subjected to hotspot panel-genomic sequencing (GeneseeqOne^TM^ pan-cancer 425-gene panel, Geneseeq Technology Inc.) at the coverage depth of 1500×. Finally, a total of 126 tissue samples and 41 prior-operation and 38 post-operation cfDNA samples from 53 patients were included in the analysis (Supplementary Data 2). We integrated the clinical data and the Snapshot mutation data from the BLCS cohort (https://sites.sph.harvard.edu/blcs/), who were primarily recruited in Massachusetts General Hospital and enrolled a total of 496 T_1_N_0_M_0_ stage LUAD patients for further prognosis analysis (Supplementary Fig. 1a**;** Supplementary Data 7). TCGA data were queried from the GDC data portal (https://portal.gdc.cancer.gov)^29^.

### Targeted next-generation sequencing and data processing

DNA from peripheral blood mononuclear cells (PBMCs) of the same patients served as a germline DNA reference. Peripheral blood (5–10 mL) was collected from each patient in EDTA-coated tubes (BD Biosciences). Plasma was extracted within 2 h of blood collection and shipped to the central testing laboratory within 48 h. Genomic DNA from FFPE sections or biopsy samples and whole blood samples were extracted with a QIAamp DNA FFPE Tissue kit and DNeasy Blood and tissue kit (Qiagen, USA), respectively. Circulating cell-free DNA (cfDNA) from plasma was extracted using the QIAamp Circulating Nucleic Acid kit (Qiagen). Sequencing libraries were prepared using the KAPA Hyper Prep Kit (KAPA Biosystems) according to the manufacturer’s instructions for different sample types. The target-enriched library was then sequenced on the HiSeq4000 NGS platform (Illumina) according to the manufacturer’s instructions.

In brief, Trimmomatic^30^ (v0.36) was used for FASTQ file quality control. Paired-end reads were then aligned to the reference human genome GRCh37 (https://www.ncbi.nlm.nih.gov/genome/) using the Burrows–Wheeler aligner (BWA)^31^. PCR deduplication was performed using Picard, and local realignment around indels and base quality score recalibration were performed using the Genome Analysis Toolkit (GATK v3.2)^32^. Furthermore, somatic single nucleotide variant (SNV) and insertion/deletions (INDELs) calling was performed using the Mutect2 mode of GATK. All mutations were manually inspected using the Integrative Genomics Viewer (IGV)^33^.

### Determination of copy number variation (CNV)

CNV analysis was performed using Control-FREEC^34^, which indicated CNV gain or loss for genes within panel coverage. Sequenza^35^ (v2.1.2) was used to estimate the total copy number (CNt) and allele-specific copy number (CN_A_ and CN_B_) profiles in the gene locus, then we defined the high genomic amplification (CNt ≥ 6)^36^ and gene loss (CNt = 0). TSGs were defined according to previous reports^37^, and LOH events (CN_B_ = 0) were screened for TSGs^38^. The results are shown in Supplementary Data 5 and  6.

### Construction of phylogenetic tree and determination of driver events

We derived phylogenies for each set of micro-dissected MPNs using Treeomics (v1.8.1)^39^ to estimate the truncal and branching alterations. Each phylogeny was rooted at the pre-invasive and adjacent invasive components using the Treeomics algorithm, which used the Bayesian inference model and determined the probability that a variant was either present or absent. The somatic alterations were considered truncal events when the present probabilities in two components were both >99.9%, and the somatic alterations with present probabilities >99.9% in only one component were identified as branching events. In addition, mutations with low variant allele frequency (VAF) would be excluded during phylogenetic processing for a low level of confidence. The driver alterations in trunk and branches were annotated and adjusted using driver gene and Cancer Gene Census (v84) annotation parameters within the Treeomics program.

### Tumor-infiltrating lymphocytes (TILs)

To analyze inflammatory infiltration in the TCGA cohort, we queried gene expression data from the GDC data portal (https://portal.gdc.cancer.gov)^29^. Then we applied the tumor immune cell deconvolution method TIMER^40^ to predict TILs. Six types of immune infiltrates were estimated (B cell, CD4+ T cell, CD8+ T cell, neutrophil, macrophage, and dendritic cell), and B cells and T cells were considered into further analyses. Immunohistochemistry was used to validate the infiltration of B cells and T cells using the expression of CD20 (M075501; Dako, CA, USA) and CD3 (A045201; Dako, CA, USA), respectively. Truncal *EGFR*-mutated cases were selected to test B cell and T cell infiltration in the preinvasive and invasive components of MPN, and two pathologists independently estimated the results. The final results are presented as the median number of tumor-infiltrating cells in the three randomly selected hotspot areas.

### ITH and d*N*/d*S* estimation

We estimated the ITH using the mutant-allele tumor heterogeneity (MATH) method^41^. The MATH score was calculated using the formula ${\rm{{MATH}}}_i = \frac{{{\rm{{MAD}}}({\rm{{VAF}}}_i)}}{{{\rm{{Median}}}({\rm{{VAF}}}_i)}} \times 100$, where VAF_*i*_ is a vector of the VAF of all mutations from sample *i* and median absolute deviation (MAD) was denoted. A constant factor (1.4826) was used to scale MAD such that the expected MAD of a sample from a normal distribution equaled the standard deviation. To estimate the selective pressure in each group, dndscv (https://github.com/im3sanger/dndscv)^42^ was used to compute the relative ratio of nonsynonymous to synonymous mutations (d*N*/d*S* ratio), and the calculation was only used full-length covered genes within panels.

### Oncogenic signaling pathway annotation and mutually exclusive analysis

According to the previous study^43^, somatic alterations in tumors were classified into canonical pathways, RTK-RAS, TP53, PI-3-kinase/Akt, SWI-SNF, cell cycle, Nrf2, β-catenin/Wnt, Notch, and Myc, to reveal the potential mechanisms and patterns of truncal and branching mutations. To distinguish among recurrent driver alterations, mutually exclusive analysis was performed separately on truncal driver variations in the JSCH cohort, and driver mutations in BLCS and TCGA cohorts. Pair-wise Fisher’s exact test was used in the “somaticInteractions” function of R package Maftools^44^.

### Statistical analysis and figures

Statistical analyses were performed using R (v3.5.1). For comparisons of continuous variables between groups, Mann–Whitney *U* tests and Kruskal–Wallis *H* tests were used. For comparisons of categorical variables between groups, chi-squared or Fisher’s exact tests were employed. To compare survival between groups, we used the log-rank test. All reported *P* values were two-sided. The differences were considered significant when the *P* value was <0.05. Other figures were generated using the R package ggplot2 and RColorBrewer.

### Reporting summary

Further information on research design is available in the Nature Research Reporting Summary linked to this article.

## Supplementary information

Supplementary Information

Description of Additional Supplementary Files

Supplementary Data 1

Supplementary Data 2

Supplementary Data 3

Supplementary Data 4

Supplementary Data 6

Supplementary Data 6

Supplementary Data 7

Supplementary Data 8

Reporting Summary

## Data Availability

The sequencing data reported in the study have been deposited in the EGA database as EGAD00001006457. The data is deposited under controlled access for access to the data contact Dr. Rong Yin, rong_yin@njmu.edu.cn. Data that support the findings of this study are available from BLCS (https://sites.sph.harvard.edu/blcs/) and TCGA database (https://portal.gdc.cancer.gov). All the other data supporting the findings of this study are available within supplementary files and from the corresponding author upon reasonable request.
