# Supplementary material for: Tumor evolutionary trajectories during the acquisition of invasiveness in early stage lung adenocarcinoma
Source: Nat Commun. 2020 Nov 27;11:6083. doi: 10.1038/s41467-020-19855-x (PMC7695730; doi:10.1038/s41467-020-19855-x)
Supplement: Supplementary file 1 — Supplementary Information [file 41467_2020_19855_MOESM1_ESM.pdf]

Supplementary information

Tumor evolutionary trajectories during the acquisition of invasiveness in early stage lung adenocarcinoma

Siwei Wang; Mulong Du; Jingyuan Zhang; Weizhang, Xu; Qianyu Yuan; Ming Li; Jie Wang; Hongyu Zhu; Yuzhuo Wang; Cheng Wang; Yuhua Gong; Xiaonan Wang; Zhibin Hu; David C. Christiani; Lin Xu; Hongbing Shen; Rong Yin.

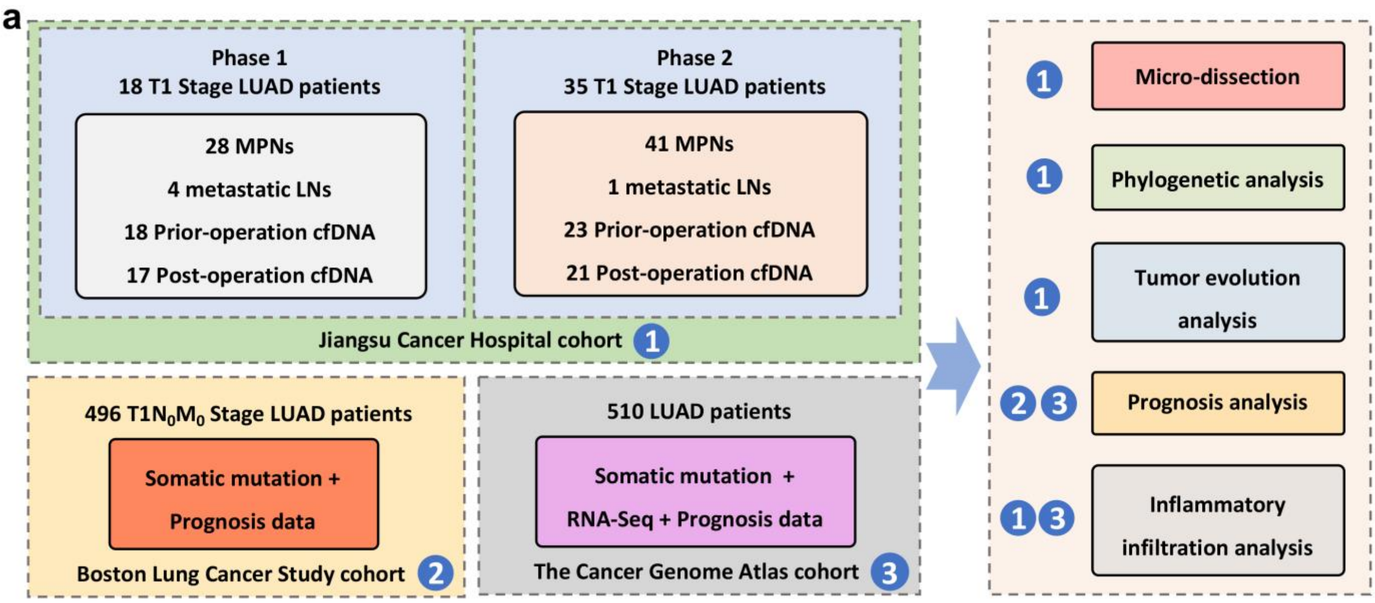
a
Phase 1
18 T1 Stage LUAD patients
28 MPNs
4 metastatic LNs
18 Prior-operation cfDNA
17 Post-operation cfDNA
Phase 2
35 T1 Stage LUAD patients
41 MPNs
1 metastatic LNs
23 Prior-operation cfDNA
21 Post-operation cfDNA
Jiangsu Cancer Hospital cohort 1
496 $T1N_0M_0$ Stage LUAD patients
Somatic mutation + Prognosis data
Boston Lung Cancer Study cohort 2
510 LUAD patients
Somatic mutation + RNA-Seq + Prognosis data
The Cancer Genome Atlas cohort 3
1 Micro-dissection
1 Phylogenetic analysis
1 Tumor evolution analysis
2 3 Prognosis analysis
1 3 Inflammatory infiltration analysis

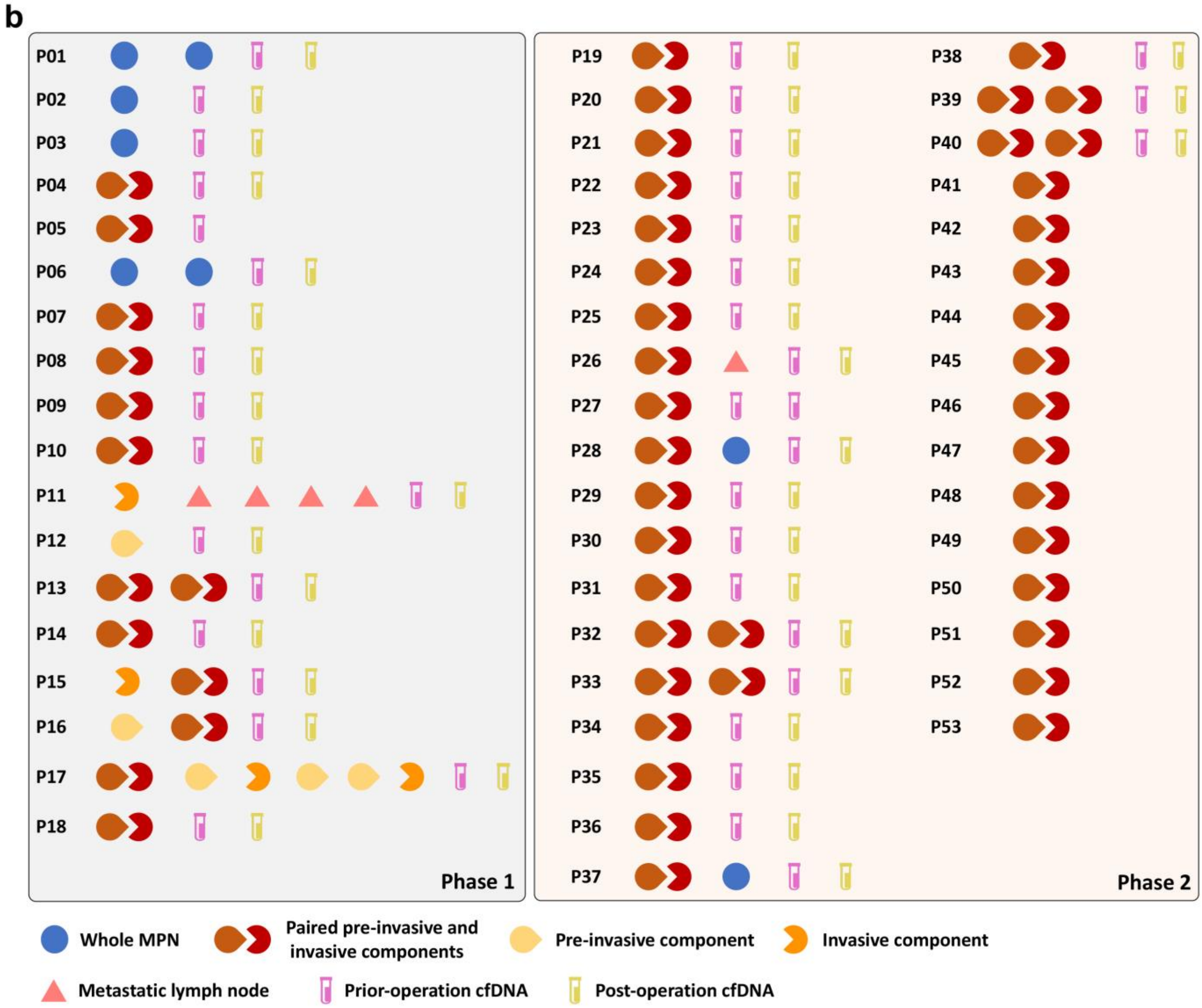

Supplementary Fig. 1 General diagram of data and workflow for this study. a A total of 53 T1 stage LUAD patients were included in the two-phase studies, which were performed with genomic sequencing using a 1021-gene panel (depth of ~1800×) and 425-gene panel (depth of ~1500×) separately. The Boston Lung Cancer Study (BLCS) cohort and the Cancer Genome Atlas (TCGA) data were also included in the following analyses. **b** In this two-phase study of the JSCH cohort, a total of 113 MPN components, 8 whole MPNs, 5 metastatic lymph nodes (MLNs), 79 cfDNA and matched 53 peripheral blood specimens were performed with genomic sequencing.

a

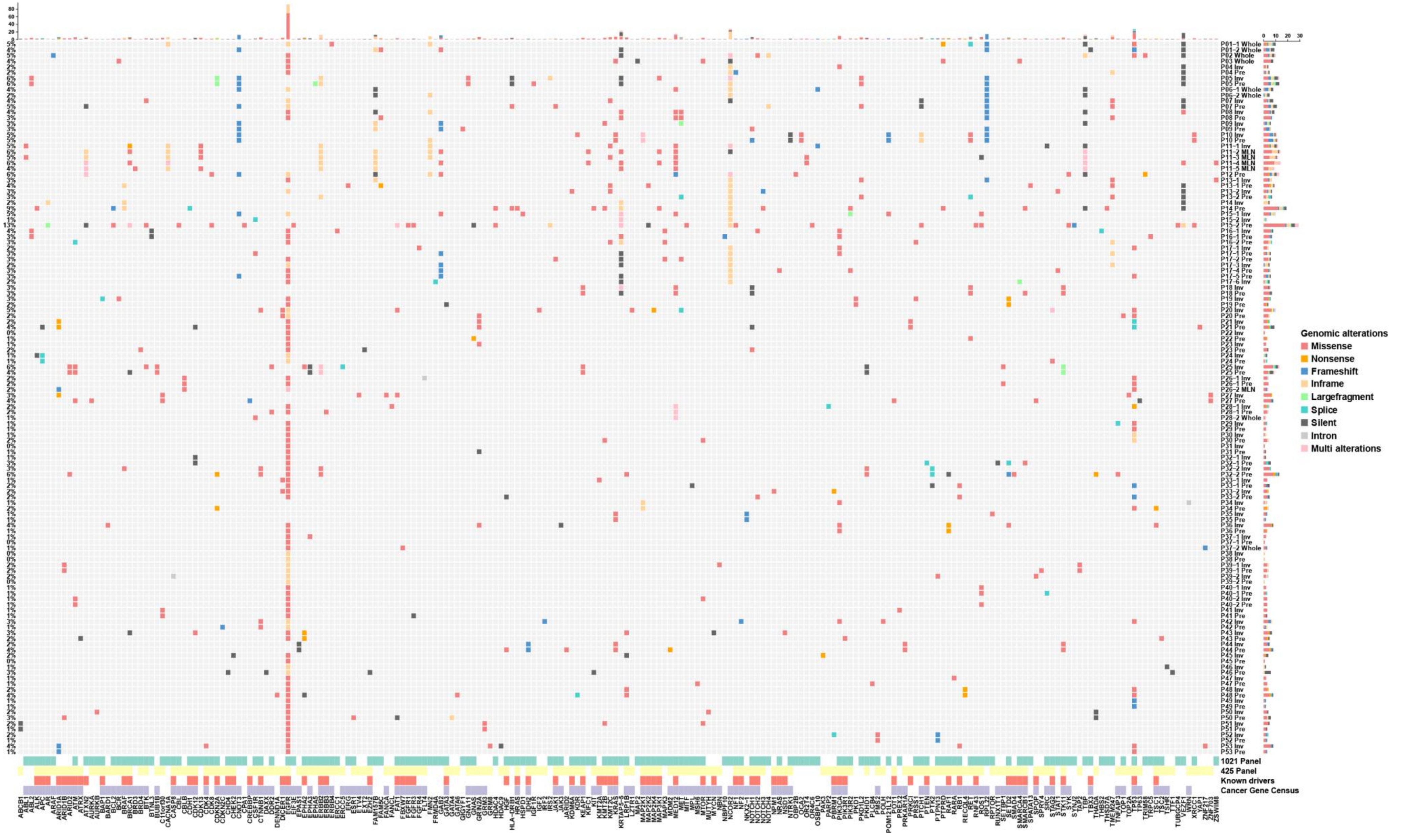
Genomic alterations
Missense
Nonsense
Frameshift
Inframe
Largefragment
Splice
Silent
Intron
Multi alterations
1021 Panel
425 Panel
Known drivers
Cancer Gene Census

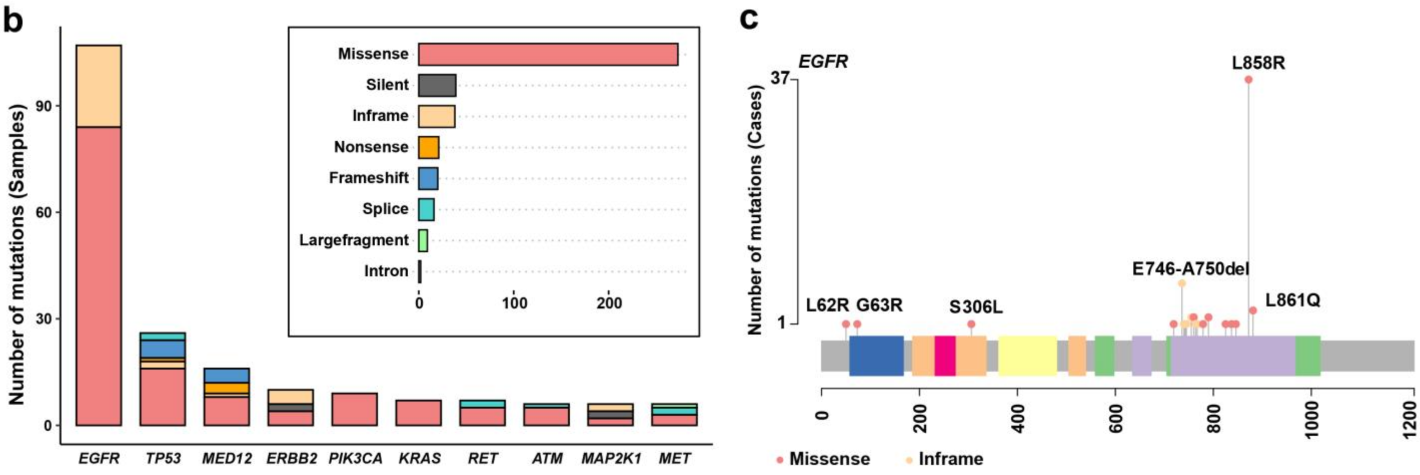

Supplementary Fig. 2 Landscape of somatic mutations in tissue samples. a Genomic variants detected by targeted next-generation sequencing of tissue samples, including MPNs and lymph nodes, in the included 53 patients. Most of mutated genes were known drivers (Methods). **b** Significant mutated driver genes and somatic variant classification among all included 121 MPN tissue samples. **c** Lolliplot indicates recurrent pathogenic alterations of *EGFR*. Inv, Invasive; Pre, Pre-invasive; Whole, Whole MPN; MLN, Metastatic lymph nodes.

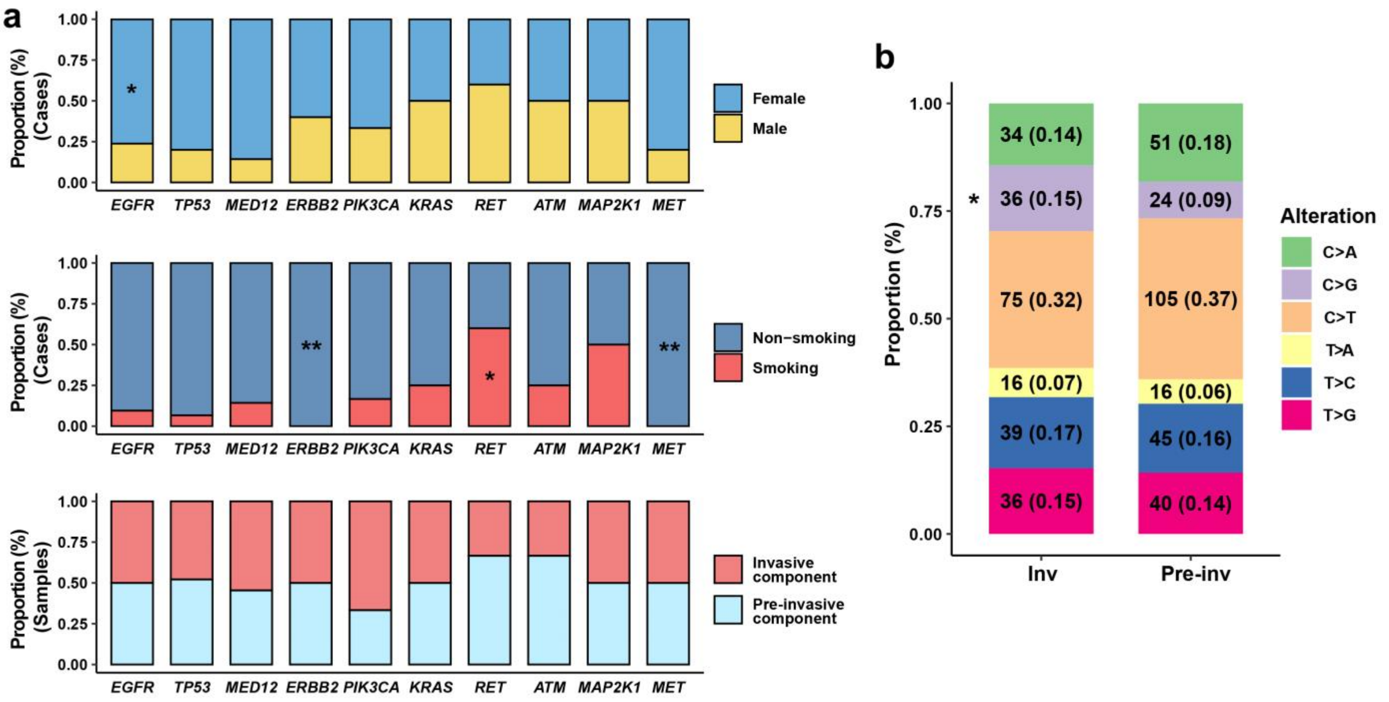

Supplementary Fig. 3 Characteristics of somatic mutations in invasive and pre-invasive MPN components. a Comparison of the top mutated driver genes according to gender and smoking status among all 53 cases. No significant differences in driver genes were observed between pre-invasive and invasive MPN components (Pre-invasive, $n = 57$; Invasive, $n = 56$). Differential proportions were compared using two-sided Fisher's exact test, with corresponding P values of 0.025 (*EGFR* in male vs. female), as well as 0.002, 0.013, and 0.002 (*ERBB2*, *RET*, and *MET* in non-smokers vs. smokers), respectively. **b** Six mutational subtypes in 57 pre-invasive and 56 invasive MPN components were assessed by two-sided Fisher's exact test ($P = 0.019$). Inv, Invasive and Pre-Inv, Pre-invasive. $^{**}P < 0.01$; $^{*}P < 0.05$.

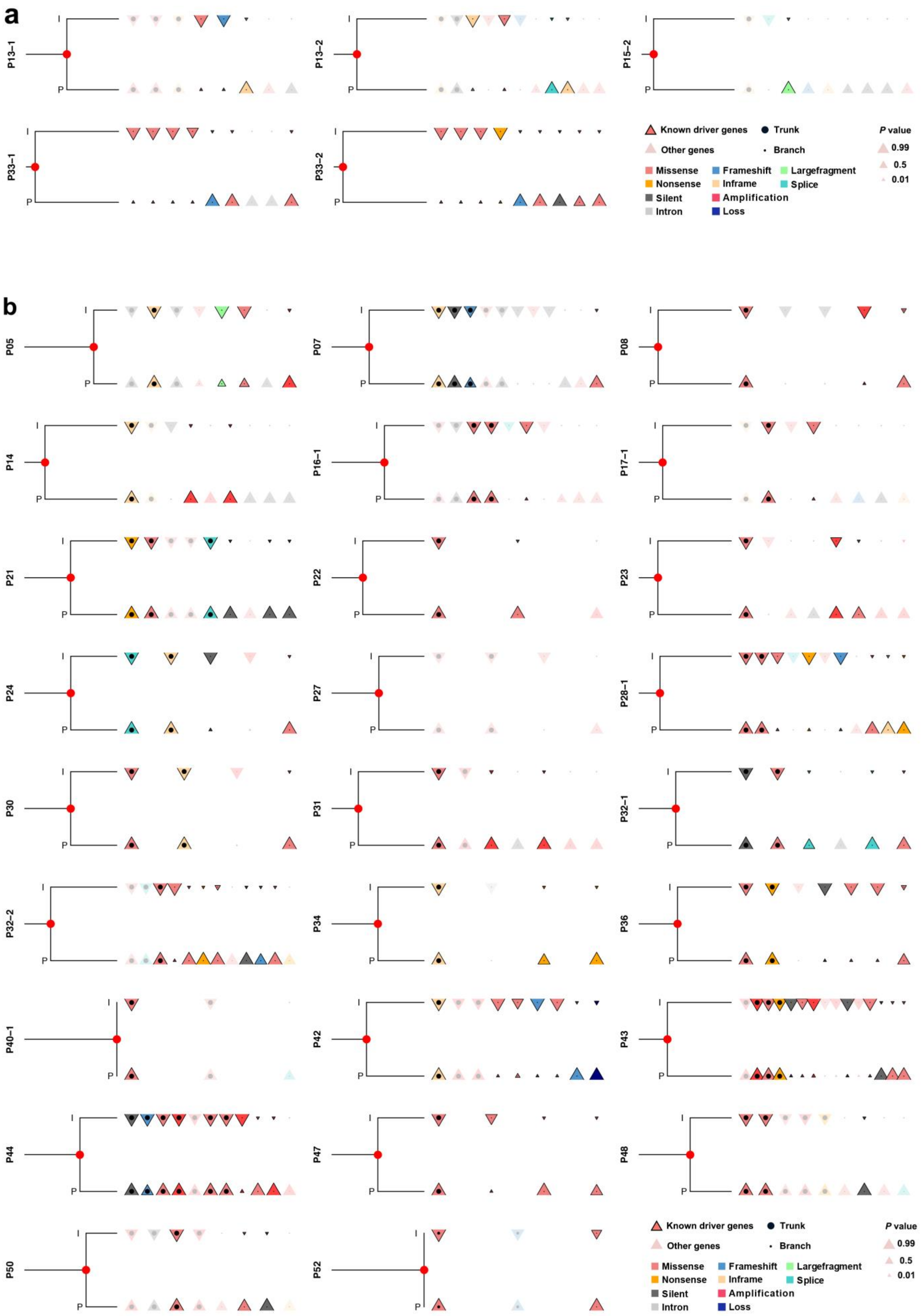
a
P13−1
P13−2
P15−2
P33−1
P33−2
Known driver genes
Other genes
Trunk
Branch
P value
0.99
0.5
0.01
Missense
Nonsense
Silent
Intron
Frameshift
Inframe
Amplification
Loss
Largefragment
Splice
b
P05
P07
P08
P14
P16−1
P17−1
P21
P22
P23
P24
P27
P28−1
P30
P31
P32−1
P32−2
P34
P36
P40−1
P42
P43
P44
P47
P48
P50
P52

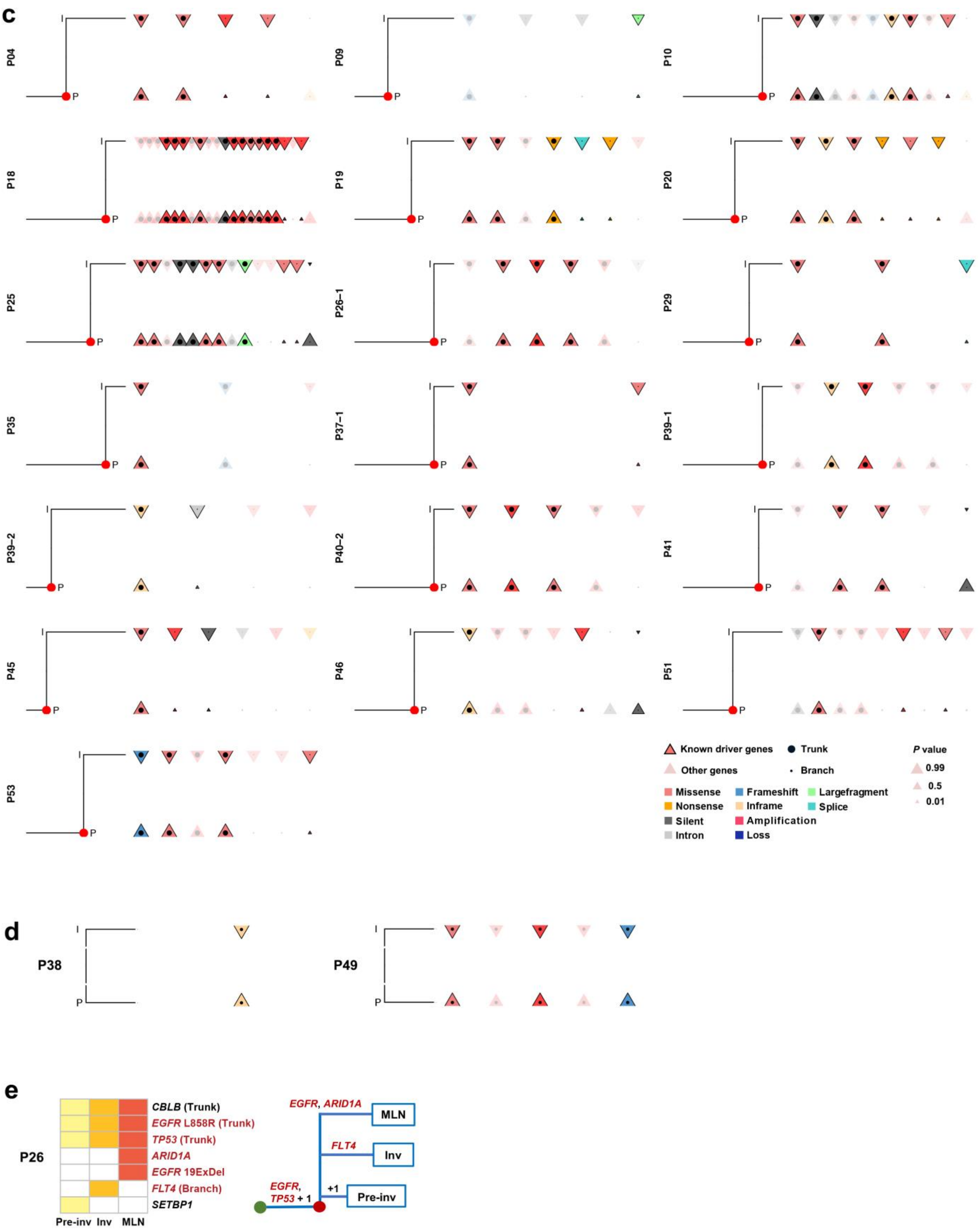
c
P04
P09
P10
P18
P19
P20
P25
P26–1
P29
P35
P37–1
P39–1
P39–2
P40–2
P41
P45
P46
P51
P53
Known driver genes
Other genes
Trunk
Branch
P value
0.99
0.5
0.01
Missense
Nonsense
Silent
Intron
Frameshift
Inframe
Amplification
Loss
Largefragment
Splice
d
P38
P49
e
P26
CBLB (Trunk)
EGFR L858R (Trunk)
TP53 (Trunk)
ARID1A
EGFR 19ExDel
FLT4 (Branch)
SETBP1
Pre-inv
Inv
MLN
EGFR, ARID1A
FLT4
EGFR, TP53 + 1
+1

Supplementary Fig. 4 Phylogenetic analyses on 52 paired MPN components (Supplementary table 4). **a** Five sets of MPN components of evolution mode 1 (EM1). No shared driver events between pre-invasive and adjacent invasive components. **b** Twenty-six sets of MPN components of EM2A. Pre-invasive components harbor branching key alterations. **c** Nineteen sets of MPN components of EM2B. Critical variations are restricted to invasive components. **d** No different genetic variations were detected in the two MPNs, which therefore could not be classified into any EMs. The size of the triangle indicates the probability that the variant is present or absent, which suggests truncal or branching variations (Methods). **e** Phylogenetic analysis on somatic mutations of lymph node metastatic JSCH P26 ($T1N1M_0$; EM2B) indicated the linear evolution model among pre-invasive component, invasive component and MLN. P/Pre-inv, Pre-invasive; I/Inv, Invasive.

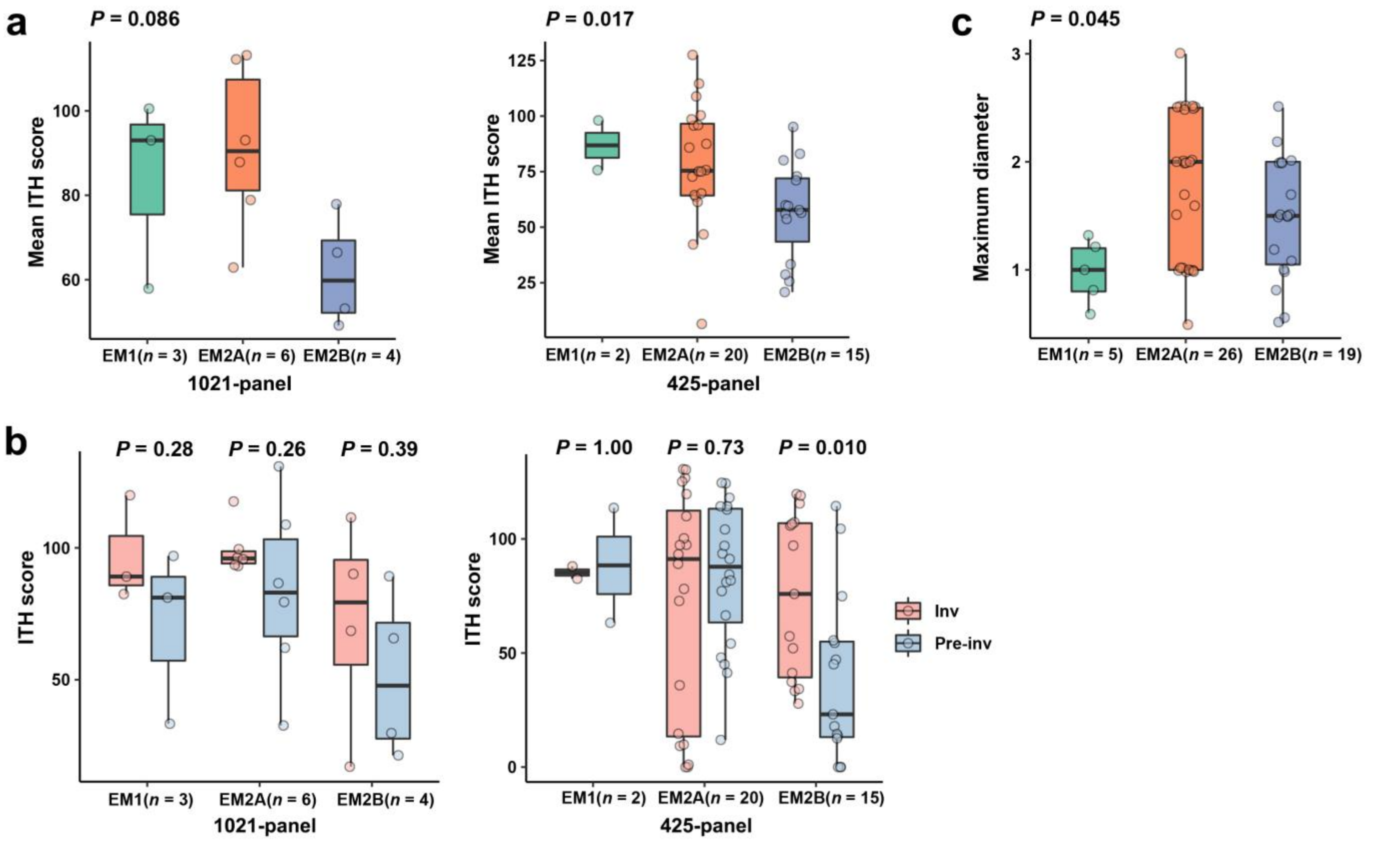

Supplementary Fig. 5 Phenotypic differences among the three evolution modes. a The mean intratumor heterogeneity (ITH) score of pre-invasive and adjacent invasive components in the phase 1 and 2 studies separately. The differences among EMs were assessed using the Kruskal–Wallis H test. **b** Comparisons of ITH levels between paired pre-invasive and invasive components using two-sided Wilcoxon Rank-Sum test. For EM2B, the trend of increased ITH level in invasive components was consistently observed in two panels. **c** Differential tumor sizes among three EMs by Kruskal–Wallis H test. Bar, median; box, 25th to 75th percentile (Interquartile range, IQR); vertical line, data within 1.5 times the IQR. Pre-inv, Pre-invasive; Inv, Invasive.

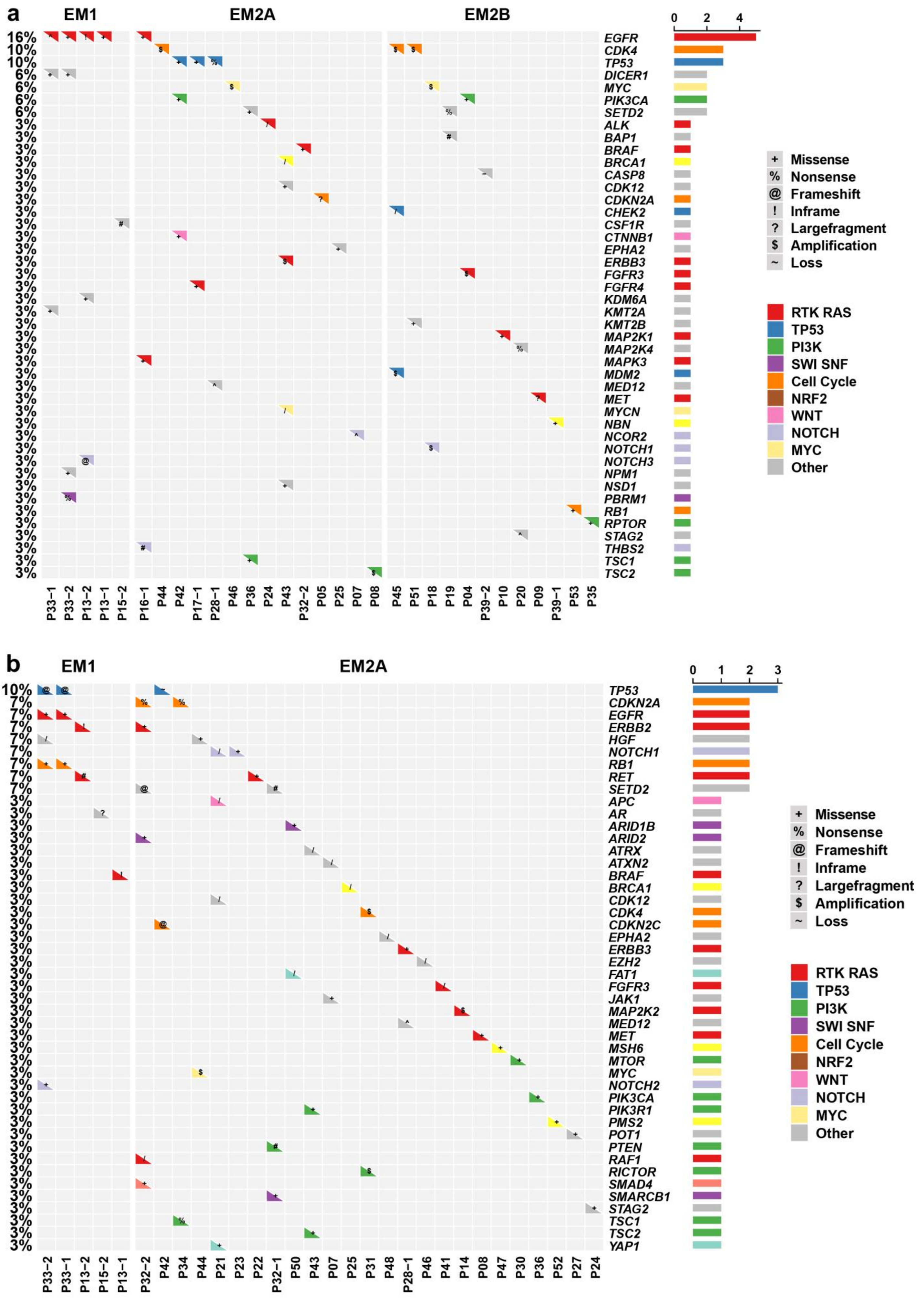
a
EM1
EM2A
EM2B
0 2 4
EGFR
CDK4
TP53
DICER1
MYC
PIK3CA
SETD2
ALK
BAP1
BRAF
BRCA1
CASP8
CDK12
CDKN2A
CHEK2
CSF1R
CTNNB1
EPHA2
ERBB3
FGFR3
FGFR4
KDM6A
KMT2A
KMT2B
MAP2K1
MAP2K4
MAPK3
MDM2
MED12
MET
MYCN
NBN
NCOR2
NOTCH1
NOTCH3
NPM1
NSD1
PBRM1
RB1
RPTOR
STAG2
THBS2
TSC1
TSC2
16%
10%
10%
6%
6%
6%
6%
3%
P33−1 P33−2 P13−2 P13−1 P15−2 P16−1 P44 P42 P17−1 P28−1 P46 P36 P24 P43 P32−2 P05 P25 P07 P08 P45 P51 P18 P19 P04 P39−2 P10 P20 P09 P39−1 P53 P35
+ Missense
% Nonsense
@ Frameshift
! Inframe
? Largefragment
$ Amplification
~ Loss
RTK RAS
TP53
PI3K
SWI SNF
Cell Cycle
NRF2
WNT
NOTCH
MYC
Other
b
EM1
EM2A
0 1 2 3
TP53
CDKN2A
EGFR
ERBB2
HGF
NOTCH1
RB1
RET
SETD2
APC
AR
ARID1B
ARID2
ATRX
ATXN2
BRAF
BRCA1
CDK12
CDK4
CDKN2C
EPHA2
ERBB3
EZH2
FAT1
FGFR3
JAK1
MAP2K2
MED12
MET
MSH6
MTOR
MYC
NOTCH2
PIK3CA
PIK3R1
PMS2
POT1
PTEN
RAF1
RICTOR
SMAD4
SMARCB1
STAG2
TSC1
TSC2
YAP1
10%
7%
3%
P33−2 P33−1 P13−2 P15−2 P13−1 P32−2 P42 P34 P44 P21 P23 P22 P32−1 P50 P43 P07 P25 P31 P48 P28−1 P46 P41 P14 P08 P47 P30 P36 P52 P27 P24
+ Missense
% Nonsense
@ Frameshift
! Inframe
? Largefragment
$ Amplification
~ Loss
RTK RAS
TP53
PI3K
SWI SNF
Cell Cycle
NRF2
WNT
NOTCH
MYC
Other

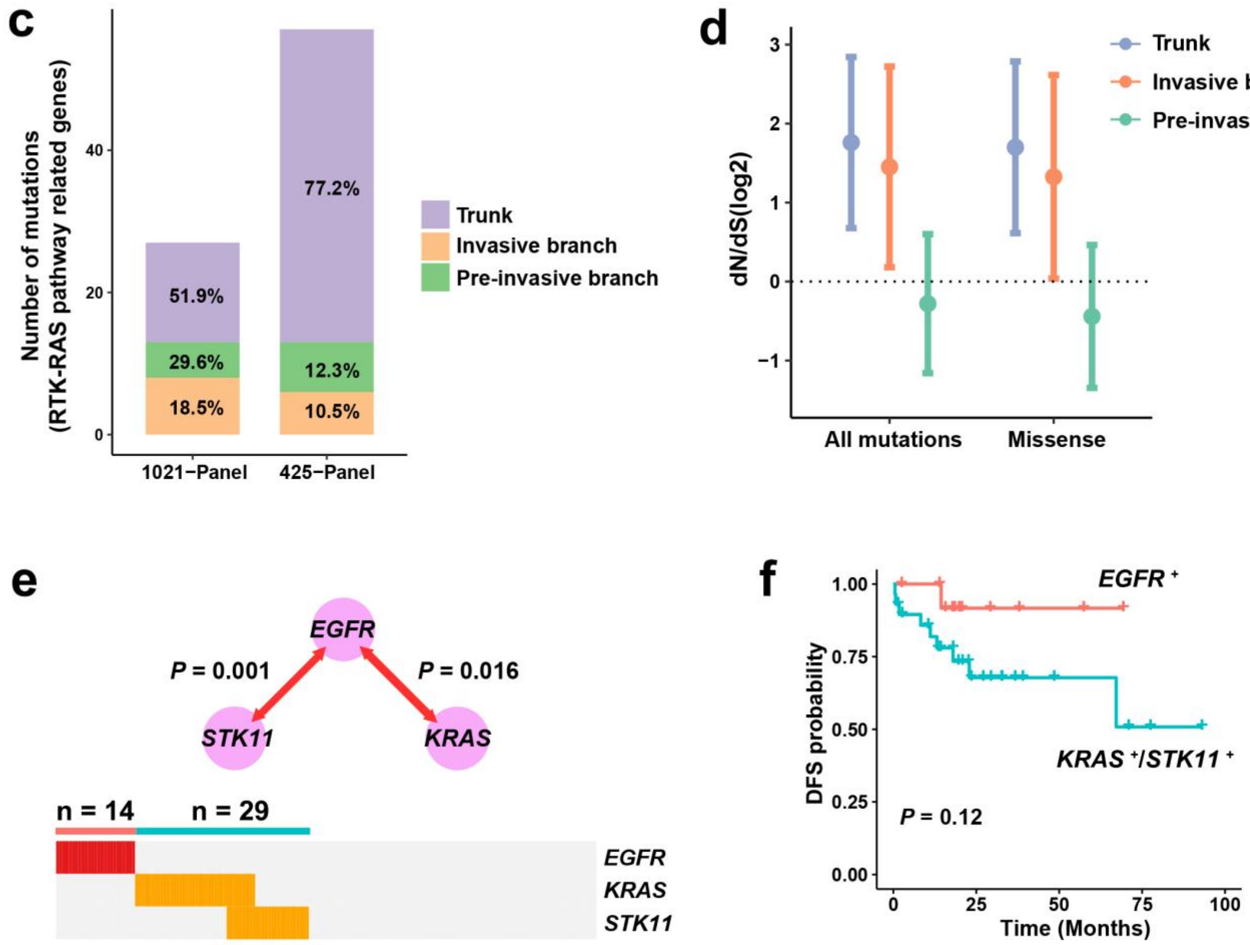

Supplementary Fig. 6 Characteristics of invasive and pre-invasive branch alterations. a,b Mutational spectrum of recurrent branching invasive (**a**) and pre-invasive alterations (**b**). Branching genes were defined according to known oncogenic pathways identical to those previously reported (Methods). **c** Proportions of truncal and branching mutations in RTK-RAS pathway genes (MPNs of 1021-panel, *n* = 13; MPNs of 425-panel, *n* = 39). Two-phase results both indicated that RTK-RAS pathway-related variations contributed mostly to truncal mutations. **d** The ratios of nonsynonymous to synonymous mutations among trunks, pre-invasive and invasive branches in the 425-panel data only (MPNs, *n* = 39). These ratios were obtained as described for **Fig. 3b**. Circles and vertical lines correspond to the mean and 95% confidence intervals of the dN/dS ratio, respectively. **e** Mutually exclusive analyses of driver mutations in the TCGA data. *P* value was calculated by pairwise Fisher's exact test. **f** Kaplan–Meier curves showing a trend of better prognosis for *EGFR*-mutated cases. *P* value is indicated from log-rank test. DFS, Disease-free survival.

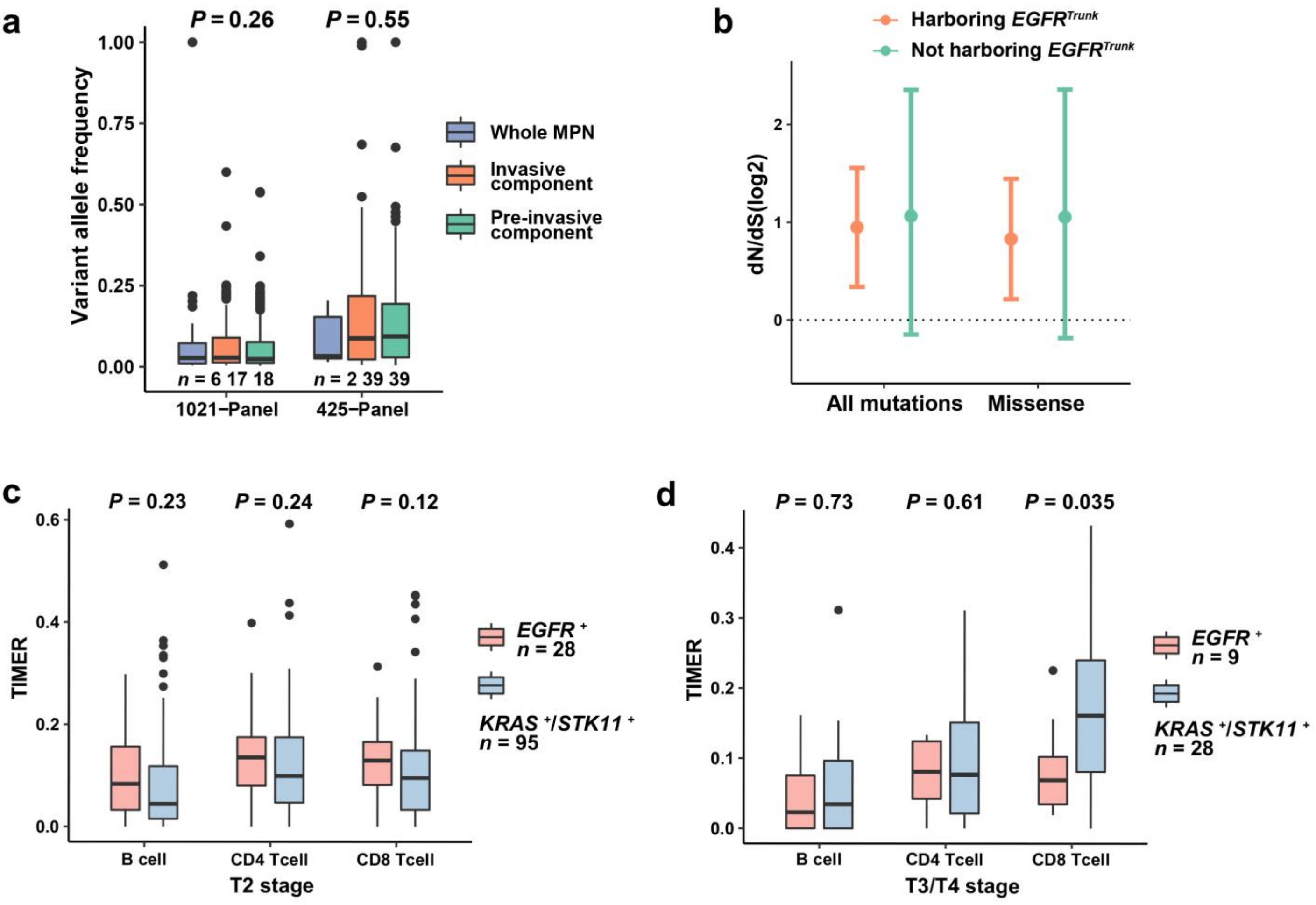

Supplementary Fig. 7 The relationship between truncal *EGFR* mutation and strong selective pressure. a No significantly differential abundance among whole MPNs, pre-invasive and invasive components in both two panels. *P* values were calculated by Kruskal–Wallis H test. **b** dN/dS ratios inferred for MPNs harboring (n = 32) or not harboring truncal (n = 5) *EGFR* mutations in the 425-panel data only. These ratios were obtained as described for **Fig. 3b**. Circles and vertical lines correspond to the mean and 95% confidence intervals of the dN/dS ratio, respectively. **c,d** Comparisons on T cells (**c**) an B cells (**d**) between *EGFR*- and *KRAS/STK11*-mutated groups using TIMER inflammatory infiltration in T2-4 stage cases of TCGA. *P* values were derived from two-sided Wilcoxon rank-sum test. The box plot displays the first and third quartiles (top and bottom of the boxes), median (band inside the boxes), and lowest and highest point within 1.5 times the interquartile range of the lower and higher quartiles (whiskers).

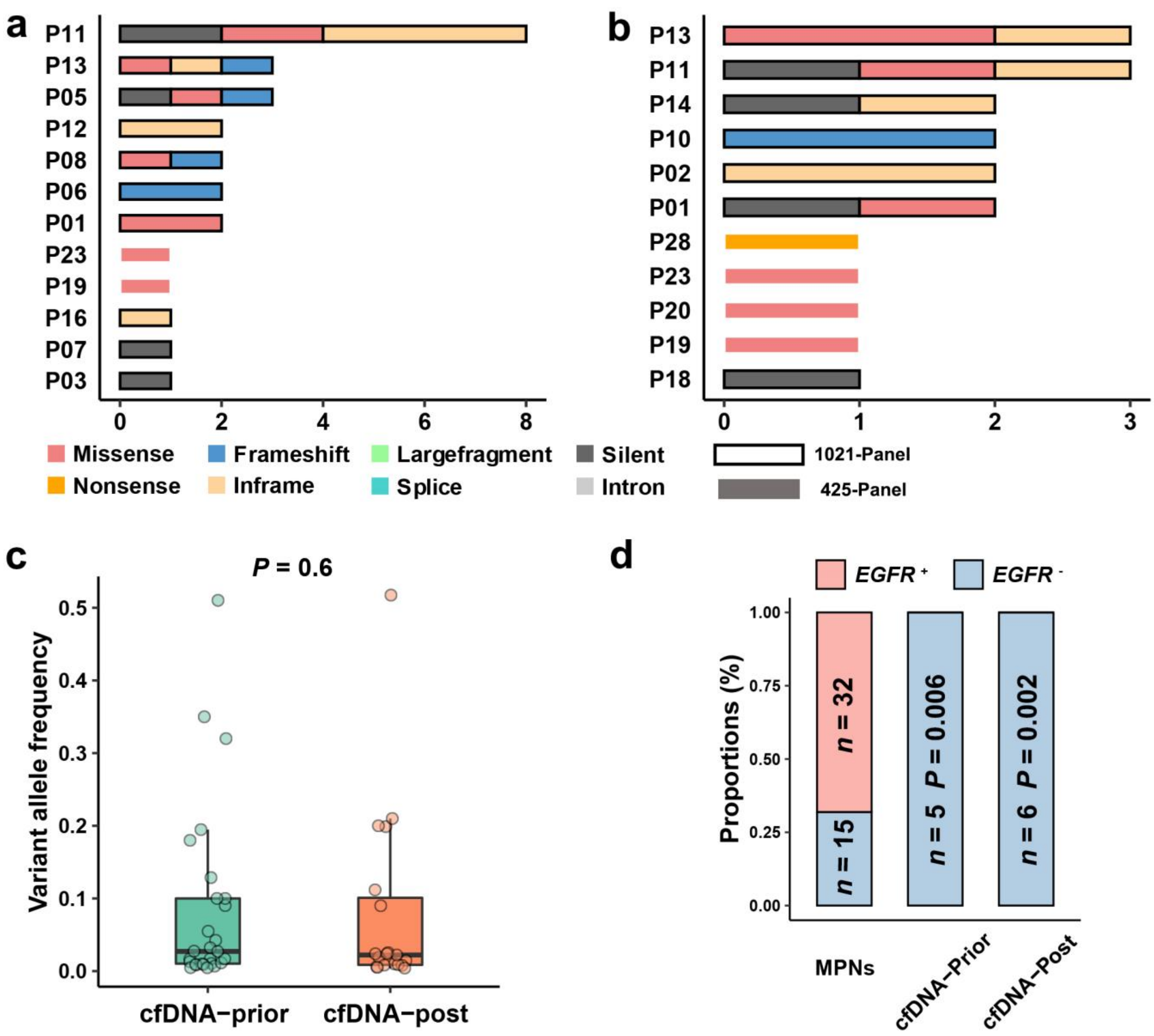

Supplementary Fig. 8 Results of analyses of cfDNAs in this T1 stage cohort. a, b The number of somatic mutations in 12 prior-operation (**a**) and 11 post-operation cfDNA samples (**b**) separately. **c** Comparison of mutation abundance between 12 prior-operation and 11 post-operation cfDNAs. *P* value, two-sided Wilcoxon rank-sum test. The box plot displays the first and third quartiles (top and bottom of the boxes), median (band inside the boxes), and lowest and highest point within 1.5 times the interquartile range of the lower and higher quartile (whiskers). **d** For cfDNAs harbored common somatic mutations with primary lesions, none of *EGFR* mutations were detected both in prior- and post-operation cfDNAs as assessed using two-sided Fisher's exact test.
