# Supplementary material for: Tumor evolutionary trajectories during the acquisition of invasiveness in early stage lung adenocarcinoma
Source: Nat Commun. 2020 Nov 27;11:6083. doi: 10.1038/s41467-020-19855-x (PMC7695730; doi:10.1038/s41467-020-19855-x)
Supplement: Supplementary file 2 — Description of Additional Supplementary Files [file 41467_2020_19855_MOESM2_ESM.pdf]

Description of Additional Supplementary Files

File Name: Supplementary Data 1
Description: Clinicopathologic characteristics of the T1 stage LUAD patients JSCH cohort.

File Name: Supplementary Data 2
Description: The list of included samples with gene-panel sequencing.

File Name: Supplementary Data 3
Description: All somatic mutations identified in this cohort study by gene-panel sequencing.

File Name: Supplementary Data 4
Description: Phylogenetic results of somatic variants using Treeomics inference

File Name: Supplementary Data 5
Description: Copy number related critical events identified in tissue samples.

File Name: Supplementary Data 6
Description: Homozygotic mutation and loss of heterozygosity plus mutation of tumor suppressor genes in this cohort.

File Name: Supplementary Data 7
Description: Clinicopathologic characteristics of included cases of BLCS cohort.

File Name: Supplementary Data 8
Description: Comparison of EGFR mutations between cfDNA samples and corresponding MPNs.
