# Supplementary material for: Tumor evolutionary trajectories during the acquisition of invasiveness in early stage lung adenocarcinoma
Source: Nat Commun. 2020 Nov 27;11:6083. doi: 10.1038/s41467-020-19855-x (PMC7695730; doi:10.1038/s41467-020-19855-x)
Supplement: Supplementary file 11 — Reporting Summary [file 41467_2020_19855_MOESM11_ESM.pdf]

nature research

Corresponding author(s): Rong Yin

Last updated by author(s): Aug 11, 2020

# Reporting Summary

Nature Research wishes to improve the reproducibility of the work that we publish. This form provides structure for consistency and transparency in reporting. For further information on Nature Research policies, see Authors & Referees and the Editorial Policy Checklist .

## Statistics

For all statistical analyses, confirm that the following items are present in the figure legend, table legend, main text, or Methods section.

| n/a | Confirmed | |
|---|---|---|
| ☐ | ☒ | The exact sample size (*n*) for each experimental group/condition, given as a discrete number and unit of measurement |
| ☐ | ☒ | A statement on whether measurements were taken from distinct samples or whether the same sample was measured repeatedly |
| ☐ | ☒ | The statistical test(s) used AND whether they are one- or two-sided<br>*Only common tests should be described solely by name; describe more complex techniques in the Methods section.* |
| ☐ | ☒ | A description of all covariates tested |
| ☐ | ☒ | A description of any assumptions or corrections, such as tests of normality and adjustment for multiple comparisons |
| ☐ | ☒ | A full description of the statistical parameters including central tendency (e.g. means) or other basic estimates (e.g. regression coefficient) AND variation (e.g. standard deviation) or associated estimates of uncertainty (e.g. confidence intervals) |
| ☐ | ☒ | For null hypothesis testing, the test statistic (e.g. *F*, *t*, *r*) with confidence intervals, effect sizes, degrees of freedom and *P* value noted<br>*Give P values as exact values whenever suitable.* |
| ☐ | ☒ | For Bayesian analysis, information on the choice of priors and Markov chain Monte Carlo settings |
| ☐ | ☒ | For hierarchical and complex designs, identification of the appropriate level for tests and full reporting of outcomes |
| ☒ | ☐ | Estimates of effect sizes (e.g. Cohen's *d*, Pearson's *r*), indicating how they were calculated |

*Our web collection on statistics for biologists contains articles on many of the points above.*

## Software and code

Policy information about availability of computer code

| | |
|---|---|
| Data collection | No software was used for data collection. |
| Data analysis | Trimmomatic (v0.36), BWA (v 0.7.12), Picard (v1.119), GATK (v3.2), IGV(v2.7), Control-FREEC (v11.5), Sequenza (v2.1.2), dndscv (v 0.1.0) and Treeomics (v1.8.1). All statistic analyses were performed in R (v3.5.1). |

For manuscripts utilizing custom algorithms or software that are central to the research but not yet described in published literature, software must be made available to editors/reviewers. We strongly encourage code deposition in a community repository (e.g. GitHub). See the Nature Research guidelines for submitting code & software for further information.

## Data

Policy information about availability of data

All manuscripts must include a data availability statement. This statement should provide the following information, where applicable:

- Accession codes, unique identifiers, or web links for publicly available datasets
- A list of figures that have associated raw data
- A description of any restrictions on data availability

The sequencing data reported in the study have been deposited in the Genome Sequence Archive for Human (http://bigd.big.ac.cn/gsa-human/) at the BIG Data Center, Beijing Institute of Genomics, Chinese Academy of Sciences, under accession number PRJCA003452. All the other data supporting the findings of this study are available within supplementary files and from the corresponding author upon reasonable request.

# Field-specific reporting

Please select the one below that is the best fit for your research. If you are not sure, read the appropriate sections before making your selection.

# Life sciences study design

All studies must disclose on these points even when the disclosure is negative.

| | |
|---|---|
| Sample size | No statistical methods were used to predetermine sample size. We retrieved high-quality panel-sequencing data from 53 T1 stage lung adenocarcinoma patients, including 113 MPN components, 8 whole MPNs, 5 metastatic lymph nodes, 79 cfDNA and matched 53 peripheral blood specimens (Supplementary Fig. 1b; Supplementary Data 2). The number of samples included was based on the number of samples available for analysis from ChiCTR1900022521 cohort. |
| Data exclusions | We excluded one post-operation cfDNA sample (P05 patient) that failed sequencing. |
| Replication | No replication was applied to any individual sample. Fifteen formalin fixed paraffin-embedded (FFPE) sections of each MPN were prepared for the micro-dissection and extracted genomic DNA for the panel-sequencing. |
| Randomization | This was an observational study, no randomization was performed. |
| Blinding | Blinding was not considered appropriate for this study. |

# Reporting for specific materials, systems and methods

We require information from authors about some types of materials, experimental systems and methods used in many studies. Here, indicate whether each material, system or method listed is relevant to your study. If you are not sure if a list item applies to your research, read the appropriate section before selecting a response.

## Materials & experimental systems

| n/a | Involved in the study | |
|---|---|---|
| ☐ | ☒ | Antibodies |
| ☒ | ☐ | Eukaryotic cell lines |
| ☒ | ☐ | Palaeontology |
| ☒ | ☐ | Animals and other organisms |
| ☐ | ☒ | Human research participants |
| ☐ | ☒ | Clinical data |

## Methods

| n/a | Involved in the study | |
|---|---|---|
| ☒ | ☐ | ChIP-seq |
| ☒ | ☐ | Flow cytometry |
| ☒ | ☐ | MRI-based neuroimaging |

## Antibodies

| | |
|---|---|
| Antibodies used | CD20 (1:100, M075501; Dako, California, USA); CD3 (1:100, A045201; Dako, California, USA) |
| Validation | The antibody was purchased from Dako which provided a rigorous protocol for antibody validation. Validation statements of primary antibodies on manufacturer's websites and applications: https://www.agilent.com/cs/library/brochures/00036_primary_ab_list_row_version.pdf |

## Human research participants

Policy information about studies involving human research participants

| | |
|---|---|
| Population characteristics | All patients were from China (Supplementary Data 1) and USA (Supplementary Data 7). All patients presented as malignant pulmonary nodules and treated with surgical resection. |
| Recruitment | No patients were recruited particularly for this study. Patients were treated as standard of care and resected specimens were analyzed. |
| Ethics oversight | Written informed consent was obtained from all patients involved. The study was approved by the institutional review board of Jiangsu Cancer Hospital. |

Note that full information on the approval of the study protocol must also be provided in the manuscript.

## Clinical data

Policy information about clinical studies

All manuscripts should comply with the ICMJEguidelines for publication of clinical research and a completedCONSORT checklist must be included with all submissions.

| | |
|---|---|
| Clinical trial registration | ChiCTR1900022521 |

| | |
|---|---|
| Study protocol | The purpose of this study is to investigate the temporal heterogeneity of genomic variation in patients with resected non-small cell lung cancer (NSCLC) by comparing the spatial heterogeneity of genomic variation within the same lesion, between primary and metastatic lesions, and between tissue and plasma samples, which was further monitored with plasma ctDNA. Finally, we attempt to establish the evolution model of lung cancer based on large data and clinical information. Full protocal can be accessed in http://www.chictr.org.cn/showproj.aspx?proj=34204. |
| Data collection | This observational study was conducted at Jiangsu Cancer Hospital (Nanjing, China) from April 2017 through Aprial 2024. |
| Outcomes | The primary and secondary endpoints of the study was progression free survival (PFS) and overall survival (OS). PFS was defined as the time from the beginning of therapy to disease progression, or the time to last follow up for patients that were progression-free (patients that were progression-free on the date of last follow up were censored on that date). OS was defined as the time from the beginning of therapy to death, or last follow-up date for patients who were alive (the latter were censored on that date). |
